# Comparison of mean pain scores for the patients with sub hepatic drainage to those without it after elective uncomplicated laparoscopic cholecystectomy

**DOI:** 10.12669/pjms.35.1.224

**Published:** 2019

**Authors:** Faisal Nadeem, Muhammad Rehan Khan, Fahim Ullah Naz

**Affiliations:** 1 Dr. Faisal Nadeem, FCPS. Department of Surgery, Military Hospital, Rawalpindi, Pakistan; 2 Dr. Muhammad Rehan khan, resident surgery. Department of Surgery, Military Hospital, Rawalpindi, Pakistan; 3 Dr. Faheem Ullah Naz, resident surgery. Department of Surgery, Military Hospital, Rawalpindi, Pakistan

**Keywords:** Mean pain score, Visual analogue scale, Sub hepatic drain, Laparoscopic cholecystectomy

## Abstract

**Objective:**

The sub hepatic drain is often placed after laparoscopic cholecystectomy is considered to affect post operative infection, pain and sub hepatic collections. The objective of this study was to compare the degree of postoperative pain in patients with routine drainage with those without it after elective, uncomplicated laparoscopic cholecystectomy.

**Methods:**

This randomized control trial (RCT) was done over six months from 9^th^ June 2015 to 8^th^ December 2015 at Military Hospital Rawalpindi. Sample calculated with WHO calculator and consecutive non probability random sampling used to divide 170 patients undergoing laparoscopic cholecystectomy in two groups. One group had routine sub hepatic drain and other didn’t. Degree of postoperative pain was assessed according to VISUAL ANALOGUE SCALE by duty doctor at 24 hours. Data was collected and analyzed applying chi square test and p value was <.05 considered statistically significant.

**Results:**

Our results demonstrated that intensity of post operative pain in routine drainage group is higher as compared to non drainage group after elective, uncomplicated laparoscopic cholecystectomy.

**Conclusion:**

Routine placement of sub hepatic drain in elective uncomplicated laparoscopic cholecystectomy should be avoided to reduce post-operative pain.

## INTRODUCTION

Cholelithiasis is a common problem among masses and two to three percent of asymptomatic patients become symptomatic each year.^1^ Laparoscopic cholecystectomy was introduced as an alternative to conventional open gallbladder removal, by Mouret in 1987 and it soon became gold standard for the surgical treatment of cholelithiasis.^2^,^3^ Role of routine sub hepatic drainage after Laparoscopic cholecystectomy is still an issue of great debate.^4^,^5^ An intra-abdominal drainage inserted as an early warning system may not always detect a nearby fluid collection and it also poses risk of liver, vascular and potentially a visceral injury. It is also a potential entry site for microorganisms and has pain during its removal.^6^ Sub hepatic drain is a constant source of irritation and pain for the patient. The aim of the drainage of the sub hepatic space after removal of gall bladder is also to avoid bile or blood accumulation that may become infected and require interventional treatment, either by use of imaging or possibly surgical approach.

In a study conducted by Shamim M et al postoperative pain was 18.99% in drain group and 5.66% in group without drainage.^7^ In one of the studies conducted, the morbidity rate was lower in no drain group (odds ratio-1.97, confidence interval 95 %, P 0.003). Other studies fail to show significant difference between the two groups.^8^

The rationale of the study was to assess the severity of postoperative pain in drain group and compare it with non drain group to carry out analysis and apply it in our routine work. It would direct us to counter postoperative pain if cause seems to be routine sub hepatic drainage.

## METHODS

Our study was carried out at Department of Surgery Military Hospital Rawalpindi for a period of six months from June 9^th^ to December 8^th^ 2015 and was a randomized controlled trial. Our sample calculated by WHO calculator, came as 170 with 85 in group one and 85 in group two. Level of significance was 5% and Power of the test was 80% with anticipated P1 and P2 at 18.99 and 5.66 respectively. Inclusion criteria comprised of all patients undergoing elective laparoscopic cholecystectomy including both genders. Age limit of 30-55 years was applied. Exclusion criteria comprised of those patients converted to open cholecystectomy, Immunocompromised and requiring critical care. It also included Patients with neuralgias and with complications like post operative hemorrhage or biliary leakage.

After approval of the study by the institution’s research ethics committee, the patients meeting inclusion criteria were enrolled in the study after explaining the procedure to them in the language they understood and informed written consent was taken. Demographic information like age, gender and address was endorsed in a predesigned Performa and telephone numbers were also taken for follow up. Consecutive non-probability sampling technique was applied and the patients undergoing elective laparoscopic cholecystectomy, meeting inclusion criteria were assigned randomly to one of the two groups. Group one received sub hepatic drain during the operation and group two had no sub hepatic drainage. Degree of post operative pain was assessed according to Visual Analogue Scale by duty doctor at 24 hours in both groups. Patients developing complications such as biliary leak and hematoma were omitted from the study.

Data was recorded on separate forms. Data collected through Performa was entered into SPSS version 17.0 and analyzed through its statistical package. Descriptive statistics were used to calculate quantitative and qualitative variables. Qualitative variables like gender were measured in frequency percentages. Quantitative variable, pain was measured as mean pain score with VAS. Chi square test was applied to compare frequency of pain between the two groups. P value of ≤0.05 was considered significant. Effect modifiers like age gender were controlled by stratification. Ethical issues were maintained by informing about factors such as data coding, disposal, sharing and archiving.

## RESULTS

Age distribution of the patients was done showing that 15.29%(n=26) were between 30-35 years of age, 18.82% (32) were between 36 to 40 years, 10.58% (n=18) between 41 to 45 years of age, 22.35% n= 38 between 46 – 50 years and 32.94% n=56 between 51 to 55 years of age, mean ± SD was calculated as 46 ± 7.65 years for Group-I and 47±7.63 years for Group-II years.

Patients were distributed according to gender, it shows that 21.17%(n=36) were males while 78.82%(n=134) were females of the total sample. In Group-I females were 38.82%(n=66) and males were 11.17%(n=19). In Group-II females were 39.41%(n=68) and males were 10% (n=17).

**Table-I:** Frequencies of post operative pain (n=170).

Post operative Pain	Group A	Group B	Total	P value
Yes	18 (21.17%)	5 (5.88%)	23 (13.52%)	*0.0035*
No	67 (78.82%)	80 (94.11%)	147 (86.47%)	

Total	85 (100%)	85 (100%)	170(100%)	

Frequency of significant pain score in Group-I was 21.17% n=18 and frequency of significant pain score in Group-II was 5.88% n=5. The combined frequency of significant pain score in both groups combined was 13.52 % n=23. P value was 0.0035.

## DISCUSSION

Cholelithiasis is a major problem in the western population and also in our part of the world. It has been traditionally treated with open cholecystectomy. Routine drainage of the sub hepatic space has been a surgical trend of open cholecystectomy, carried on to the era of laparoscopic surgery without substantial evidence. The sub hepatic drain is placed to drain blood, bile and serous fluid from that space and is an early warning system for detection of operative complications such as blood collection or biliary leak. In a study done by Antoniou S, Pain scores were significantly higher in the drainage group both at 6-12h (mean difference 1.12, 95% CI 1.01-1.24, P<0.0001) and at 12-24h after surgery (mean difference 1.12, 95% CI 0.86-1.39, P<0.0001). This was also comparable with our results where significant pain was higher in drain group (p=0.0035).^9^ Sub hepatic drainage is considered to increase infection, pain and sub hepatic collections.^10^,^11^

Laparoscopic cholecystectomy (LC) has largely replaced conventional cholecystectomy in the past decade and is the gold standard now.^12^,^13^ However, there are still limited data about the value of prophylactic sub-hepatic drainage for elective uncomplicated LC. Pain is a major concern for patients undergoing laparoscopic cholecystectomy.^14^,^15^ The studies carried out to ascertain this difference have revealed variable results. Our study was carried out to determine the frequency of pain score in patients with drain group as compared to those without it, in uncomplicated laparoscopic cholecystectomy.

Our study shows that 21.17 %(n=36) were male while 78.82%(n=134) were females. Frequency of significant pain score in Group-I was 21.17% n=18 and frequency of significant pain score in Group-II was 5.88% n=5. The frequency of significant pain score in both groups combined was 13.52% n=23. P value was 0.0035 indicating significant statistical difference. Pain in the drain group was significantly higher after observation at 24 hours.

The results of our study were similar to study done by Shamim M, published in Indian journal of surgery. According to his study significant pain was 18.99% in drain group comparable to our result of 21.17% in drain group, whereas his results for significant pain were 5.66%, very close to our 5.88% in non drain group.

Results of majority of the studies conducted in different populations of the world in establishing role of sub hepatic drain in laparoscopic cholecystectomy patients revealed that it is associated with significant postoperative pain, increases discomfort as well as hospital stay for the patients.^16^,^17^,^18^ Laparoscopic cholecystectomy is primarily aimed at reducing patient’s pain, hospital stay and allows the patient a smooth and quick recovery from the operation.^19^,^20^ Sub hepatic drainage appears to be source of significant pain and this pain hinders the primary objective of the surgery which is to have quick and complication free recovery.

We are of the opinion that routine placement of sub hepatic drain after laparoscopic cholecystectomy should be restrained and discouraged to allow the patients smooth and pain free recovery.

### Limitations

Our patients were not followed in long term for pain and other long term complications such as adhesions, port site hernias and intestinal obstruction. Study was done in military set up and targeted specific class of patients. Moreover it was one at Rawalpindi so patients in this particular area were enrolled and results may not be generalized for entire Pakistani population.

## CONCLUSION

The results of our study are in accordance with the majority of studies on this subject and we concluded that the frequency of post operative pain in routine drainage group after elective laparoscopic cholecystectomy is higher as compared to non drainage group and routine placement of sub hepatic drain in elective uncomplicated laparoscopic cholecystectomy should be avoided to reduce post operative pain. However, post operative complications such as sub hepatic abscess, hematoma and bilioma may alter the outcome.

### Authors Contribution

**FN:** Conceived, designed, analyzed data and prepared manuscript.

**MRK:** Edited manuscript did literature search.

**FUN:** Performed data collection and proof reading of manuscript.
